# Automated Clinical Grade Expansion of Regulatory T Cells in a Fully Closed System

**DOI:** 10.3389/fimmu.2019.00038

**Published:** 2019-02-01

**Authors:** José Manuel Marín Morales, Nadine Münch, Katja Peter, Daniel Freund, Uta Oelschlägel, Kristina Hölig, Thea Böhm, Anne-Christine Flach, Jörg Keßler, Ezio Bonifacio, Martin Bornhäuser, Anke Fuchs

**Affiliations:** ^1^GMP Facility, DFG-Center for Regenerative Therapies Dresden, Center for Molecular and Cellular Bioengeneering, Technische Universität Dresden, Dresden, Germany; ^2^Department of Hematology, Medical Clinic I, University Hospital Carl Gustav Carus, Technische Universität Dresden, Dresden, Germany; ^3^Miltenyi Biotec GmbH, Bergisch Gladbach, Germany; ^4^DFG-Center for Regenerative Therapies Dresden, Center for Molecular and Cellular Bioengeneering, Technische Universität Dresden, Dresden, Germany; ^5^National Center for Tumor Diseases, Dresden, Germany

**Keywords:** regulatory T cells, treg cell therapy, Treg expansion, closed system, automation, advanced therapy medicinal product, CliniMACS Prodigy

## Abstract

Adoptive transfer of T regulatory cells (Treg) has been successfully exploited in the context of graft-versus-host disease, transplantation, and autoimmune disease. For the majority of applications, clinical administration of Treg requires laborious *ex vivo* expansion and typically involves open handling for culture feeds and repetitive sampling. Here we show results from our approach to translate manual Treg manufacturing to the fully closed automated CliniMACS Prodigy® system reducing contamination risk, hands-on time, and quality variation from human intervention. Polyclonal Treg were isolated from total nucleated cells obtained through leukapheresis of healthy donors by CD8^+^ cell depletion and subsequent CD25^high^ enrichment. Treg were expanded with the CliniMACS Prodigy® device using clinical-grade cell culture medium, rapamycin, IL-2, and αCD3/αCD28 beads for 13–14 days. We successfully integrated expansion bead removal and final formulation into the automated procedure, finalizing the process with a ready to use product for bedside transfusion. Automated Treg expansion was conducted in parallel to an established manual manufacturing process using G-Rex cell culture flasks. We could prove similar expansion kinetics leading to a cell yield of up to 2.12 × 10^9^ cells with the CliniMACS Prodigy® and comparable product phenotype of >90% CD4^+^CD25^high^CD127^low^FOXP3^+^ cells that had similar *in vitro* immunosuppressive function. Efficiency of expansion bead depletion was comparable to the CliniMACS® Plus system and the final ready-to-infuse product had phenotype stability and high vitality after overnight storage. We anticipate this newly developed closed system expansion approach to be a starting point for the development of enhanced throughput clinical scale Treg manufacture, and for safe automated generation of antigen-specific Treg grafted with a chimeric antigen receptor (CAR Treg).

## Introduction

Nine years after the first in-man report, there are currently close to 30 recruiting or ongoing clinical trials administering Treg in autoimmune diseases, solid organ transplantation, pro-inflammatory diseases and graft-versus-host disease (GvHD) (1, 2). Most clinical applications require *ex vivo* expansion of Treg, classifying the cell product as advanced therapy medicinal product (ATMP). Treg expansion requires activation through the T cell receptor (TCR) in the presence of high doses of IL-2 (3–5). Efficient good manufacturing practice (GMP) compliant protocols for Treg expansion have been developed by us and others (6–18) and in the case of CliniMACS isolated Treg, typically include rapamycin as cell culture medium supplement to prevent T effector cell outgrowth (11, 15, 17, 19–22). We reported manual Treg expansion for cGvHD treatment using cell differentiation bags (Miltenyi Biotec) (18, 23) and since then have changed to G-Rex100 cell culture devices (Wilson Wolf manufacturing) due to enhanced growth rates, likely related to optimized gas exchange through the permeable membrane bottom, and convenient handling. *Ex-vivo* Treg expansion for cellular therapy typically requires 2–5 weeks depending on the starting material and desired final dose. The long culture requires multiple feeding and stimulation steps realized by open handling in the majority of manufacturing processes. In our opinion, three challenges have to be overcome to make expanded Treg an attractive seminal product for prospective controlled trials and potential market launch. First, other than the vast majority of current expansion protocols, media and cytokine feeds, cell activation, optional transduction, and quality control (QC) steps should avoid open handling to ensure product and personnel safety. Second, hands-on labor should be minimized to standardize manufacturing and reduce manufacturing costs. Third, realization of individualized cellular therapy for large patient cohorts will be feasible if we can use automated closed manufacturing systems with small footprint. Here we present the first proof-of-principle study exploiting *ex-vivo* Treg expansion in the fully closed CliniMACS Prodigy® system (Miltenyi Biotec).

## Materials and Methods

The recently published minimum information about Treg cells (MITREG) checklist was followed for the preparation of this paper (24). See http://w3id.org/ontolink/mitreg for MITREG document and checklist.

### Cell Source

Unstimulated leukapheresis containing ACD-A and heparin as anticoagulants were collected from healthy donors after informed consent at the Department of Transfusion Medicine, Medical Clinic I, Carl Gustav Carus University Hospital at TU Dresden with the use of a continuous-flow cell separator (Spectra Optia®; Terumo BCT). Peripheral blood mononuclear cells (PBMCs) used for functional assays were isolated from buffy coats by standard Ficoll (Lymphoprep™, Axis-Shield) density centrifugation as described earlier (25). Buffy coats were obtained from the Deutsches Rotes Kreuz-Blutspendedienst Nord-Ost GmbH Sachsen as a side product of red blood cell isolation for clinical use. The study included sample drawing from healthy donors with informed consent approved by the local institutional review board (EK 206082008).

### Treg Isolation

Apheresis products were stored overnight at 4°C before cell isolation on the following morning (day 0 of culture protocol). Treg cell isolation was performed as previously described (18). Briefly, Treg were isolated with clinical-grade reagents in a two-step procedure under GMP conditions with the use of the CliniMACS® Plus separation system (Miltenyi Biotec). Total leukocytes containing a maximum number of 4.0 × 10^9^ CD8^+^ cells were used as starting material, allowing the usage of a single vial of CliniMACS CD8 Reagent (Miltenyi Biotec, 275-01). After depletion of CD8^+^ cells, the intermediate product was enriched for the CD25^high^ fraction (CliniMACS CD25 Reagent, Miltenyi Biotec, 274-01). As a modification of the previously published protocol (18), two washing steps were performed after CD25 labeling.

### CD4^+^CD25^−^ T Responder Cell Isolation

CD4^+^CD25^−^ T cells were isolated from PBMCs, cryopreserved and later used as responder cells (Tresp) to test the *in vitro* function of the manufactured Treg in a proliferation-based suppression assay. CD4^+^CD25^−^ cells were enriched by research scale magnetic activated cell sorting (MACS) in a two-step process using the CD4^+^ T Cell Isolation Kit human (Miltenyi Biotec) to enrich CD4^+^ T cells by negative isolation and the CD25 MicroBeads II human (Miltenyi Biotec) to deplete Treg following the manufacturer's recommendations. The enriched CD4^+^CD25^−^ population was aliquoted into 2 ml cryotubes (Greiner Bio-One) containing 1.5 × 10^7^ cells resuspended in 1 ml of CryoStor® CS10 solution (BioLife Solutions). Vials were readily placed into a CoolCell™ LX cell freezing container (Corning) and directly transferred to a −80°C freezer. Within 3 days, cryotubes were transferred to a liquid nitrogen tank for long term storage.

### Phenotyping

Treg purity and viability was determined by flow cytometry (Canto II, BD Biosciences) from aliquots taken during isolation, during the *ex vivo* expansion protocol (day 8, 12), on the day of Treg harvest (day 13 or 14) and from the final ready-to-infuse product. Extracellular staining of 0.5–1 × 10^6^ cells was performed in 100–200 μl of PBS or CliniMACS buffer. The following antibodies from BD Biosciences were used: CD45/CD3/CD4/CD8/CD16/56/CD19 (Multitest-6 color, 337166), CD25-V450 (clone M-A251), CD14-FITC (clone M5E2), CD127 PerCP5.5 (clone HIL-7R-M21), CD3 APC-H7 (clone SK7), CD45-V500 (clone HI30), CD45RO-PE (UCHL1). eBiosciences™/Invitrogen (ThermoFisher): CD45RA-V450 (clone HI100), CD62L-PE (clone DREG56). After incubation for 15 min at room temperature in the dark, cells were washed with 2 ml PBS (500 g, 5 min) and resuspended in 500 μl for flow cytometric analysis. Intracellular staining of FOXP3 was performed using the FOXP3/Transcription Factor Staining Buffer Set (eBioscience™/Invitrogen, ThermoFisher) according to the manufacturer's recommendations and the FOXP3-PE-Cy7 conjugated antibody (clone PCH101) from the same manufacturer. Cell viability was determined by 7-AAD/AnnexinV staining using the AnnexinV Apoptosis Detection Kit eFluor™450 (eBioscience™/Invitrogen, ThermoFisher) as recommended in the datasheet.

### Manual Treg Expansion

Freshly isolated cells were seeded at 0.3–0.5 × 10^6^ cells/ml in G-Rex® 100 flasks (Wilson Wolf Manufacturing) containing TexMACS GMP Medium (Miltenyi Biotec) supplemented with 100 ng/ml MACS GMP Rapamycin (Miltenyi Biotec), 1,000 U/ml IL-2 (Proleukin S, Novartis Pharma), 5% cell therapy-grade pooled human AB serum (Blutspendedienst Tübingen, Germany) and MACS GMP ExpAct Treg beads (Miltenyi Biotec) at a cell to bead ratio of 1:4. Cells were cultured at 37°C and 5%CO_2_. Cells were counted on days 5, 8, 10, and 12 and adjusted to 0.3–0.5 × 10^6^ cells/ml (day 5, 8), 0.3–1.0 × 10^6^ cells/ml (day 10) or 0.5–1.0 × 10^6^ cells/ml (day 12) by partial (50%) media replacement. Fresh medium contained all additives except ExpAct Treg beads. Cells were re-stimulated with fresh beads at a 1:1 ratio on day 8. For direct comparison to the automated culture, manual G-Rex® 10 flask cultures containing the same isolated Treg pool at 10-fold lower cell number and volume as compared to the automated culture were performed.

### Automated Treg Expansion

Automated cell culture was performed using the CliniMACS Prodigy® System (Miltenyi Biotec) equipped with a disposable CliniMACS Prodigy tubing set TS 510 (Miltenyi Biotec), which contains the single-use CentriCult cell culture and centrifugation chamber. 31–120 × 10^6^ (mean, 56.9 × 10^6^) cells were seeded on day 0 of the culture in 80–105 ml medium. Cells were cultured under defined conditions of CO_2_ (5%) and temperature (37°C). Medium composition, partial media exchange timepoints and regimens were based on our manual SOP with the difference that cell concentrations were higher in the Prodigy® system in the second week of expansion due to volume restriction of 260 ml in the CentriCult unit. Different to the manual culture, the automated culture was agitated from day 5 to ensure optimal gas supply and for cultures showing a high expansion rate an additional media exchange was conducted on day 11. For the optimized automated protocol, re-stimulation with beads on day 8 was integrated as an automated 10 ml feed after volume reduction <100 ml. Agitation was paused for 1 h to facilitate early bead to cell contact before a fresh media feed was carried out and agitation was resumed. Automated QC sampling was realized through sampling pouches as part of the tubing set or through an additionally connected triple sampling adapter (Miltenyi Biotec).

### Removal of ExpAct Treg Beads and Final Formulation

A customized process for fully automated expansion ExpAct Treg bead removal and final formulation in 0.9% NaCl/1%HA (Human Albumin 200 g/l, Baxter) infusion solution using the CliniMACS Prodigy® system was developed together with Miltenyi Biotec and successively optimized. Treg culture in the CentriCult unit, bead removal over the column and final formulation were performed with a single CliniMACS Prodigy TS 510 tubing set. The final optimized process includes multiple washing steps to exchange the media to infusion solution, after which the cells pass the primed magnetic column in a two-stage process. The final product is harvested to a sealable cell bag (Cell differentiation bag, Miltenyi Biotec). ExpAct bead removal using the CliniMACS® Plus system was performed following the manufacturer's recommendations to compare the newly developed CliniMACS Prodigy® bead removal process with the standard procedure. Briefly, CliniMACS® Plus bead removal was performed using the CliniMACS® PBS/EDTA buffer (Miltenyi Biotec) supplemented with 0.5%HA (200 g/L, Baxter), the CliniMACS® Tubing Set LS (Miltenyi Biotec) and the software sequence Depletion 2.1.

### Quantification of Residual ExpAct Treg Beads

3 × 10^7^ cells of the final Treg product were transferred to a 15 ml tube and centrifuged (300 g, 10 min, RT). The supernatant was discarded and the cell pellet was lysed in 2 ml of distilled water, vortexed and incubated for 5 min at 37°C. Subsequently, 200 μl of a 2,000 U/ml DNase I stock solution (Roche) were added and the sample was incubated for 2 min at RT. After incubation, 6 ml of MACSQuant Washing Solution (Miltenyi Biotec) were added and the vial was incubated for 10 min at RT. After centrifugation at 1,400 g for 10 min at RT, the supernatant was carefully removed and the residual beads were resuspended in the remaining volume (<30 μl). Residuals beads were quantified using a C-Chip™ Neubauer improved disposable counting chamber (Biochrom AG) and Türk's solution (Merck Millipore) at a 1:1 dilution ratio.

### Treg Suppression Assay

Functionality of the final Treg product was assessed on the basis of the ability to suppress proliferation of allogeneic CD4^+^CD25^−^ T effector cells (responder cells, Tresp). Cryostored Tresp and aliquots of manufactured Treg (analogously frozen), were thawed, washed with 10 ml X-Vivo15 media (Lonza, 04-418Q) containing 5% serum followed by a wash with 10 ml PBS. Tresp cells were stained with eFluor670 cell proliferation dye (Invitrogen) at a final concentration of 5 μM in PBS following the manufacturer's recommendations. Manufactured Treg were stained in parallel with eFluor450 cell proliferation dye (Invitrogen) at a final concentration of 10 μM to facilitate discrimination of both subsets at the time of assay readout. After incubation for 10 min at 37°C, cells were washed twice in 10 ml of cold X-Vivo15 containing 10% AB serum and resuspended in the same, but prewarmed medium for plating. 1 × 10^5^ stained Tresp cells were seeded in a round bottom 96 well cell culture plate (ThermoFisher) and Treg were added to the Tresp cells to reach Treg:Tresp ratios of 1:32, 1:16, 1:8, 1:4, 1:2, and 1:1. αCD3/αCD28 coated beads (Dynabeads™ Human T activator, ThermoFisher) were added at a bead to total cell ratio of 1:75. In order to determine maximum proliferation of Tresp, Tresp were cultured with beads in the absence of Treg. All conditions were seeded as triplicates. The final volume was adjusted to 250 μl for all conditions. Plates were incubated at 37°C for 5 days. On day 5, the individual wells were harvested and stained for CD4-FITC (clone RPA-T4, BD Biosciences) and CD25-PE-Cy7 (clone MA251, BD Biosciences). Proliferating Tresp were defined as the percentage of eFluor670^dim^CD25^+^ cells from the total Tresp population. The mean inhibition of proliferation (% suppression) found at the different Treg:Tresp ratios was calculated as (Proliferation_Tresponly_ – Proliferation_Tresp_)/Proliferation_Tresponly_ × 100. A nonlinear fit of the percentage of suppression for each condition vs. the number of added Treg for each condition was calculated using a four parameter dose-response curve algorithm in GraphPad V6.0c (GraphPad Software Inc., California, USA). The Treg:Tresp ratio responsible for 75% of inhibition of Tresp proliferation was determined as the ratio between the EC_75_ value extrapolated from the non-linear fit and the total number of Tresp cells present in the assay (1 × 10^5^ cells).

### Statistical Analysis

Statistical analyses were conducted using Prism 7.03 GraphPad Software. Unless otherwise stated, results are reported as mean values with standard deviation (SD) and *P*-values determined by two-tailed paired or unpaired Student's *t*-test.

## Results

### High Yield Isolation of Treg From Apheresis Products

**Figure 1:**
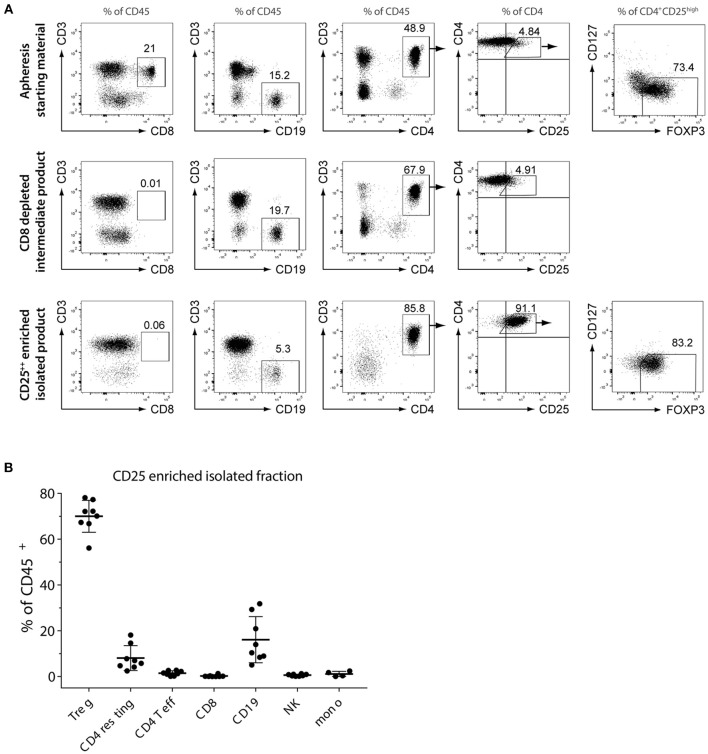
Quantification of CD4^+^CD25^high^CD127^low^FOXP3^+^ Treg and other lymphocyte subsets before and after CD8^−^CD25^high^ enrichment. **(A)** Phenotyping of initial cell source (leukapheresis), CD8^+^ depleted intermediate product and final CD25^high^ Treg enriched fraction by flow cytometry. Shown are representative flow cytometry plots for one of the donors. **(B)** Relative percentage of Treg and contaminating CD4^+^CD25^−/low^ (CD4 resting), effector memory CD4^+^CD25^+^CD127^+^CD45RO^+^ (CD4 Teff), CD8 cytotoxic T cells, B cells, NK cells and monocytes. Shown are individual and mean percentages (solid line) of CD45^+^ leukocytes of each subset for *n* = 8 Treg isolations. Error bars = SD.

CliniMACS® Treg isolation from leukapheresis through CD8 depletion and CD25 enrichment resulted in preparations with a mean purity of 72% CD4^+^CD25^high^CD127^low^FOXP3^+^ (range 56–87%, *n* = 12) and a mean yield of 61 × 10^6^ total cells (range 27 × 10^6^-155 × 10^6^, *n* = 11, data not shown). Representative flow cytometry plots before isolation, after CD8^+^ depletion and after final CD25^high^ enrichment are shown in Figure 1A. CD8^+^ depletion was highly efficient, with only mean 0.14% CD8^+^ T cells in the Treg-enriched isolated product (range 0–1.3, *n* = 16; Figure 1B). The main contaminating cells were CD25 expressing CD19^+^ B cells (mean 16.1%, range 5.1–31.8, *n* = 8), followed by CD4^+^CD25^−/low^ cells (CD4 resting) with a mean percentage of 8.07% (range 2.4–18.1, *n* = 8). Effector memory CD4^+^CD25^+^CD127^+^CD45RO^+^ (CD4 Teff) cells were present with a mean concentration of 1.47% (range 0.1–2.8, *n* = 8). NK cells expressing CD16/56 were infrequent (mean 0.62%, range 0.1–1.3, *n* = 8) and monocytes were present at a percentage of mean 1.14% (range 0.1–2,4, *n* = 4).

### Treg Manufactured by Automated Closed-System Culture Are of High Purity and Vitality

**Figure 2:**
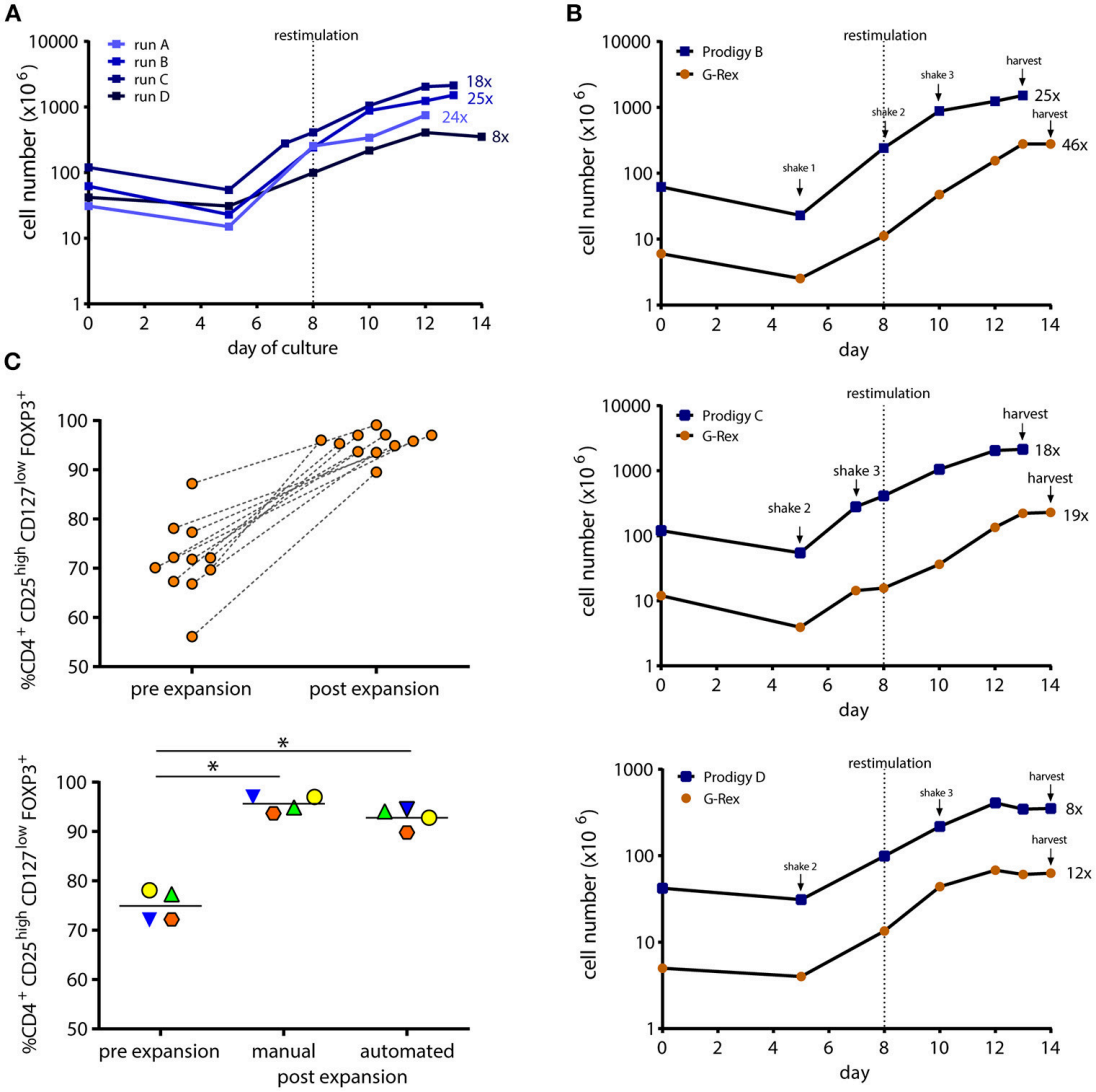
Growth curves and purity of automated closed-system vs. manually manufactured Treg. Clinical grade isolated Treg were cultured for 12–14 days in the CliniMACS Prodigy® or G-Rex10 culture devices with high dose IL-2, rapamycin, and ExpAct Treg expansion beads. **(A)** Expansion curves of four automated CliniMACS Prodigy® cultures during process development and optimization. **(B)** The optimized automated expansion process was compared to parallel manual G-Rex®10 culture starting with 1/10 of CliniMACS Prodigy® seeding cell number of the same isolated Treg pool. Bead restimulation and agitation modes are depicted. Results from 3 donors are shown. Numbers followed by x indicate fold expansion values. **(C)** Treg purity before and after manual or automated expansion culture. Treg phenotype was analyzed by flow cytometry as CD4^+^CD25^high^CD127^low^FOXP3^+^. Shown is the percentage of Treg of all CD45^+^ for manually manufactured cultures (upper panel, *n* = 11,*p < 0.05) and manual vs. CliniMACS Prodigy® expanded cultures (lower panel, *n* = 4,*p < 0.05). Matching symbols indicate same starting material.

Our standard large scale clinical-grade manual Treg expansion using G-Rex100 vessels resulted in cellular products with a Treg mean purity of 94.7% (range 89.5–98.1, *n* = 11) after 12–14 days of expansion (Figure 2C, upper panel). As published by us and other investigators, Treg expansion capacity is largely variable between donors (6, 13, 14, 17, 18, 25, 26). For direct comparison, isolated Treg enriched populations were split 1:10 after QC sampling to allow for a manual medium scale expansion culture using GRex10 devices (10 cm^2^) in parallel to each CliniMACS Prodigy® culture (100 cm^2^). We successfully optimized feeding intervals, agitation modes, media exchanges, and bead restimulation on the CliniMACS Prodigy®, reaching between 0.33 and 2.12 × 10^9^ cells on day 13 of culture with maximal cell concentrations of up to 8.5 × 10^6^ cells/ml at the time of harvest. Figure 2A shows the expansion curves for four CliniMACS Prodigy® manufacturing processes using starting material from four different donors. A lag phase seen in run A after bead restimulation was successfully diminished in later cultures through optimization of the restimulation process. Figure 2B illustrates the expansion kinetics for three optimized expansion cultures (cultures B, C, D) comparing the manual expansion in GRex10 flasks (orange symbols) to the automated expansion in the CliniMACS Prodigy® system (blue symbols). Our optimized Prodigy protocol allowed for similar kinetics as shown by the parallel expansion curves.

Treg phenotype has proven to be a critical factor for Treg stability over time. As shown by a number of investigators, loss of FOXP3 transcription factor expression occurs after prolonged *ex-vivo* culture and may correlate with reduced suppressor function (6–10, 27). The percentage of FOXP3 expressing cells within the final product is one of the release criteria in the majority of recent Treg therapy studies (13, 15, 28). We, therefore, compared Treg phenotype (CD4^+^CD25^high^CD127^low^FOXP3^+^) after manual and automated expansion cultures (Figure 2C, lower panel). At the end of culture, manually expanded Treg showed a mean purity of 95.6% (range 93.7–97.0; *n* = 4). CliniMACS Prodigy® expanded Treg of the same isolated batches were of 92.8% purity (range 89.8–94.5%, *n* = 4; *p* = 0.08). Taken together, our results indicate that the CliniMACS Prodigy® system is suitable for clinical Treg manufacturing in terms of cell yield and phenotype.

### Successful Development of Automated Expansion Bead Removal and Final Formulation

Beads coated with antibodies against CD3 and CD28 are widely used as standardized T cell stimulus mimicking both TCR engagement and costimulation by antigen presenting cells. Their use in cell manufacturing for cellular therapy requires the removal from the product prior to infusion to avoid activation of endogenous T cells. Three protocols were developed along the optimization process. As outlined in Table 1, cell recovery was improved from initially only 47% to 62% with the third developed process. Lost cells were partially detected in the CentriCult unit with initially high numbers (66 × 10^6^ cells in process II), but could be reduced by protocol optimization resulting in only 1.4–6 × 10^6^ cells in the CentriCult unit after final formulation with process III (data not shown). Residual beads in the final product were microscopically quantified from a 30 × 10^6^ cell QC sample after cell lysis and maximal volume reduction. Extrapolated residual bead content in the final product decreased to <400 beads per 100 × 10^6^ cells for the optimized process (Table 1), marking the detection limit of our bead quantification method. To directly compare the optimized CliniMACS Prodigy® bead depletion process to the widely used CliniMACS® Plus bead removal process using the Depletion 2.1 program, the two systems were directly compared. To this end, on the day of harvest, 50% of the CliniMACS Prodigy® culture volume was drawn into a cell bag as part of the tubing set by a custom partial harvest process. The collected cell fraction was bead depleted on the CliniMACS® Plus device before manual final formulation. The remainder of the culture was bead depleted using the optimized process on the CliniMACS Prodigy® system. Equal distribution of the culture was examined by cell counting. As depicted in Table 1 (frame), similar cell recovery and proportion of residual beads was observed for both methods. In conclusion, we were able to develop a custom process for the CliniMACS Prodigy® device that allows for fully automated efficient removal of ExpAct Treg expansion beads together with final formulation of the cell product, facilitating immediate bedside infusion after QC.

**Table 1:**
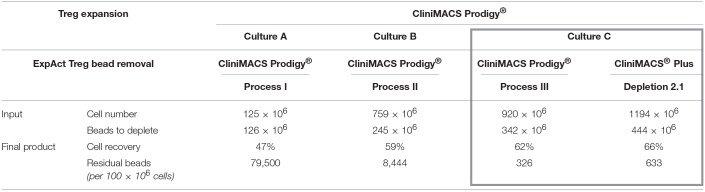
Development of expansion bead removal within the CliniMACS Prodigy® device.

*Specifications and results of three distinct customized processes for ExpAct Treg bead removal on the CliniMACS Prodigy® system. Results are shown for three individual cultures. Culture C was split by partial harvest and bead depletion was performed on the CliniMACS® Plus device in parallel to bead depletion on the CliniMACS Prodigy® system (process III). Shown are cell yield and bead removal efficiency for both devices (bold frame)*.

### CliniMACS Prodigy® Manufactured Treg Are Highly Functional

**Figure 3:**
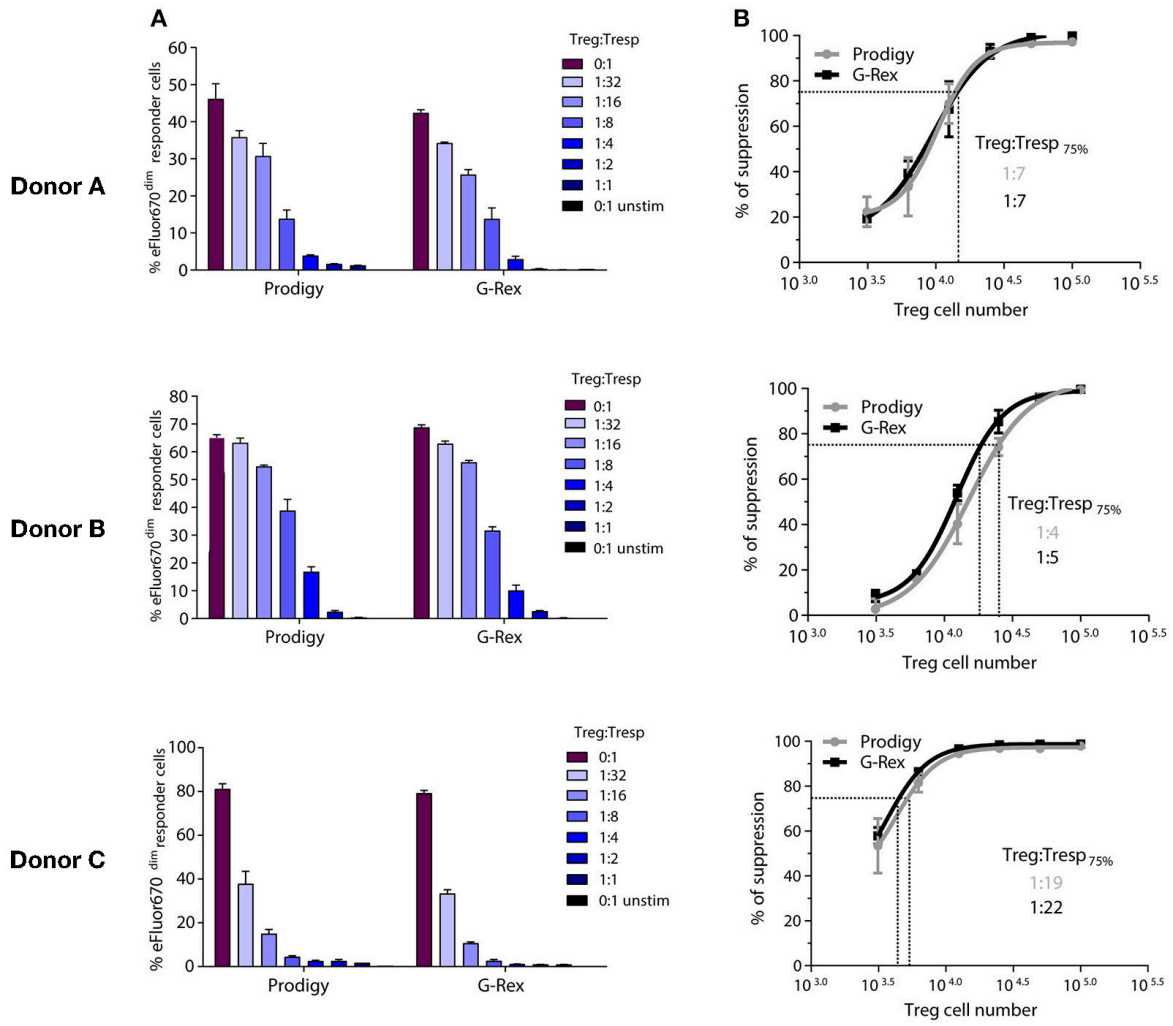
Suppressive capacity of manufactured Treg. Functionality of the final Treg products expanded by manual or automated culture was tested in a proliferation based suppression assay. **(A)** Percentage of proliferating T responder cells (Tresp) at different Treg:Tresp ratios. Each panel shows one donor. **(B)** Same data expressed as % of suppression and the estimate of the Treg:Tresp ratio required for 75% suppression. Error bars = SD.

Efficacy of Treg cellular therapy requires functionality of the final cell product. We compared the suppressive capacity of Treg manufactured by our manual vs. automated method. Functionality was measured as the ability of Treg to suppress αCD3/αCD28 bead stimulated proliferation of Treg-depleted allogeneic CD4^+^ T responder cells (Tresp) in a 5-day assay. Figure 3 shows the suppression assay results for Treg derived from three donors and expanded both by the manual and automated protocol. % of Tresp proliferation in absence and presence of increasing numbers of Treg is depicted in Figure 3A. For donor A, both CliniMACS Prodigy® and manually manufactured Treg suppressed 75% of Tresp proliferation at a Treg to Tresp ratio of 1:7 (Figure 3B). For donor B, the necessary Treg to Tresp ratio was determined as 1:4 for the CliniMACS Prodigy® manufactured Treg, and 1:5 for Treg expanded by standard manual culture. Treg from Donor C showed the highest suppressive capacity. CliniMACS Prodigy® manufactured Treg suppressed 75% of Tresp proliferation at a Tresp ratio of 1:19, whereas the respective calculated ratio for manually expanded Treg was 1:22. Suppressive capacity thus clearly varied between donors but appeared to be independent of the manufacturing regimen.

### High Stability of Final Product for Cellular Therapy

**Figure 4:**
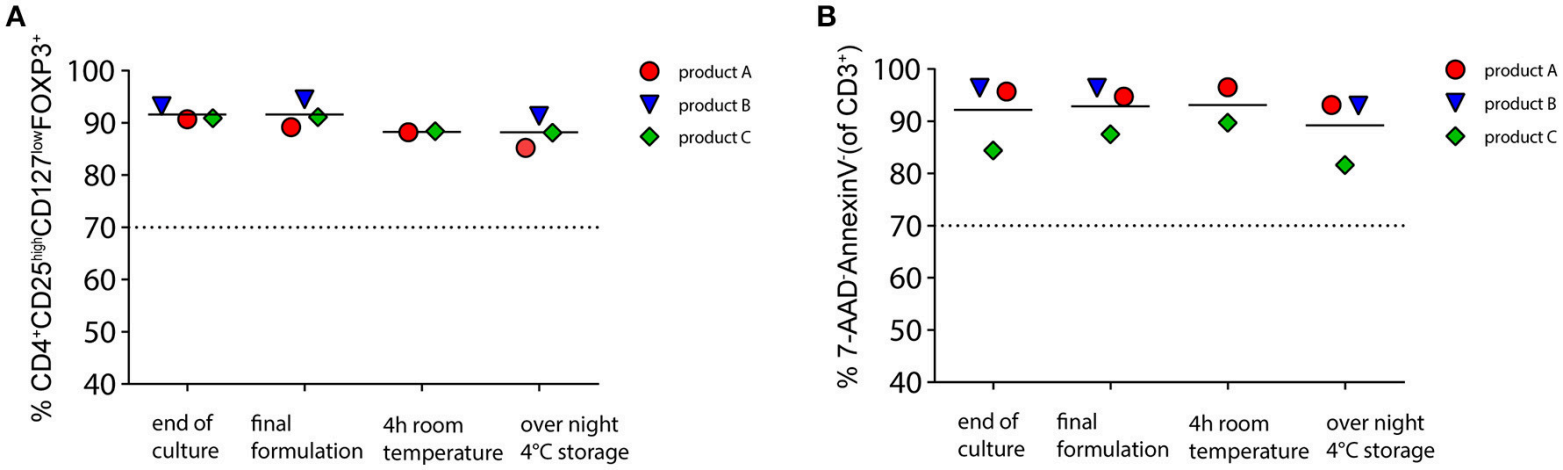
Stability of phenotype and vitality of the final cellular product. Three Treg products manufactured by automated culture were stored for 4 h in infusion solution (0.9%NaCl + 1% human albumin) at room temperature or overnight at 4°C. **(A)** Stability of Treg phenotype before and after storage as percentage of CD4^+^CD25^high^CD127^low^FOXP3^+^ of CD45^+^. **(B)** Viability of cells determined by 7-AAD/AnnexinV staining. Shown are the percentages of viable non-apoptotic cells of total CD3^+^. Broken line indicates our release criteria value.

Release relevant QC measurements for ATMP require stability of the final product over several hours. We thus determined the viability and phenotype of the CliniMACS Prodigy®-manufactured final product in infusion solution over time. As depicted in Figure 4, all three tested products were phenotypically stable with only marginal changes after overnight storage (*p* = 0.09). All products had Treg purities well above our release criteria of 70% CD4^+^CD25^high^CD127^low^FOXP3^+^ at all tested timepoints (mean at the end of culture, 91.6%; mean after overnight storage, 88.2%). The viabilities of the three products were above our release criterial of 70% 7-AAD^−^/AnnexinV^−^ live cells of total CD3^+^ at all time points. We observed a slight drop in viability after overnight storage (mean, 89.2%) as compared to the end of culture timepoint (mean, 92.2%; *p* = 0.007). Taken together, CliniMACS Prodigy® manufactured Treg can meet the release criteria for phenotype and vitality of our center also after overnight storage.

## Discussion

The development of closed cell manufacturing systems is important for the strongly growing ATMP field. Advantages over the still widely used open cell culture handling include enhanced safety for the personnel, reduced risk of product contamination, and consequently streamlined requirements by the regulatory authorities that may lower ATMP manufacturing cost in the near future. Several disposable cell culturing systems have been developed that allow closed system handling by sterile welding. Closed system Treg manufacturing in cell culture bags has successfully been developed in the context of the ONE study (17). The majority of closed systems, however, require hands-on user interaction at a similar degree to standard open cell culture systems. The CliniMACS Prodigy® system has the advantage of facilitating fully automated feeds, media exchanges, volume reduction and washing steps, reducing hands-on time and inter-operator product variability to a minimum. The device was successfully implemented for ATMP manufacturing for monocyte derived dendritic cells (29), CAR T cells (30–33) and natural killer (NK) cells (34, 35). Our proof-of-principle study is to our knowledge the first report of translating Treg manufacture to the CliniMACS Prodigy® system.

We demonstrated the feasibility of CliniMACS® Plus enrichment of Treg followed by CliniMACS Prodigy® automated Treg expansion yielding cell therapy relevant Treg numbers. Comparable to other investgators (17), Treg purity after CD8^−^CD25^+^ isolation is typically below 80% and in our hands largely dependent of the proportion of B cells as main contaminants. Purity could be enhanced by B-cell depletion as published for expansion free Treg infusion (36). However, the increase in purity to typically >90% after rapamycin supplemented expansion, indicates sufficient Treg purity after isolation by the robust CD8^−^CD25^+^ CliniMACS process. Expansion kinetics and post expansion purity of Treg manufactured with the CliniMACS Prodigy® system were comparable to the manual expansion culture, and neither phenotype nor *in vitro* function significantly differed between both tested manufacturing methods. αCD3/αCD8 coated beads are available from multiple manufacturers varying in bead material and size but with the common characteristic of being magnetic, facilitating their GMP compliant removal by the closed-system device CliniMACS® Plus. We achieved an automated CliniMACS Prodigy® based expansion bead removal process that was equally effective as the widely used bead depletion process on the CliniMACS® Plus instrument. Conveniently, the automated process could be designed to end with a readily formulated product that can be sealed off for bedside infusion. Functionality of the manufactured products was proven based on a proliferation based suppression assay. As seen by others, suppressive capacity varied between donors (17), which we hypothesize to be influenced by varying degrees of histoincompatibility of the Treg in respect to the allogeneic responder cells that were of the same batch for all assays. Importantly, suppressive capacities of Treg manufactured manually vs. automated showed comparable results for all donors. Quality control measurements are indispensable for ATMP product release. Release relevant measurements such as intracellular FOXP3 staining or the determination of residual expansion beads typically require multiple test laboratories and are time-consuming. The phenotypic and vitality stability over several hours and overnight, were, therefore, important findings which will be complemented by functional analysis data of the stored product in further manufacturing runs before regulatory approval.

A limitation of the procedure was that enrichment of Treg could not be integrated to the CliniMACS Prodigy® platform due to the unavailability of a certified CD8^+^ depletion process for the device. Using a co-localized CliniMACS® Plus to isolate Treg is, however, more cost-effective than hypothetical Treg isolation on the CliniMACS Prodigy®. The reason is that the column of the Prodigy tubing set used for culture has to be reserved for expansion bead removal, and additionally required tubing sets for isolation would be more expensive than for the CliniMACS® Plus. Our findings are based on observations and data from five consecutive runs. We acknowledge this limitation of our study and see our development of automated Treg culture as a starting point for further optimization. A substantial fraction of the product is lost during expansion bead removal and for the QC sample for quantification of residual expansion beads. Bead-free stimuli would thus be of high interest to further enhance cell product yield. Another limitation is the restriction to the 260 ml CentriCult expansion vessel, which currently remains a disadvantage over cell culture bag based processes such as the process developed by Fraser et al. (17) even though cell densities exceed those of standard vessels due to the inclusion of culture agitation. Nevertheless, market launch of tubing sets with enhanced volume CentriCult units is expected to further increase achievable cell product sizes.

Taken together, we see a clear potential for the CliniMACS Prodigy® system in standardizing polyclonal clinical Treg expansion in the future that would facilitate decentralized manufacture in large multi-center studies. We furthermore anticipate our study to be the starting point of automated antigen specific Treg manufacture by TCR or CAR transduction using the CliniMACS Prodigy ® device.

## Author Contributions

JMMM contributed to Treg manufacturing, performed experiments, contributed to data analysis and manuscript writing. NM and KP manufactured Treg. DF headed the GMP facility and critically read the manuscript. TB and A-CF contributed to manufacturing optimization and critically read the manuscript. JK developed custom application processes for the CliniMACS Prodigy device. UO supervised flow cytometry measurements. KH facilitated donor leukapheresis. EB and MB provided funding, supported the study and critically revised the manuscript. AF planned and supervised the study, contributed to Treg manufacturing, performed experiments, analyzed data, and wrote the manuscript.

### Conflict of Interest Statement

TB, A-CF, and JK are employees of Miltenyi Biotec. The remaining authors declare that the research was conducted in the absence of any commercial or financial relationships that could be construed as a potential conflict of interest.
